# Short-Term Treatment with Esmolol Reverses Left Ventricular Hypertrophy in Adult Spontaneously Hypertensive Rats via Inhibition of Akt/NF-*κ*B and NFATc4

**DOI:** 10.1155/2018/2691014

**Published:** 2018-02-18

**Authors:** Begoña Quintana-Villamandos, David A. Goukassian, Sharath P. Sasi, Emilio Delgado-Baeza

**Affiliations:** ^1^Departamento de Anestesiología, Hospital General Universitario Gregorio Marañón, Doctor Esquero 46, 28007 Madrid, Spain; ^2^Departamento de Farmacología, Facultad de Medicina, Universidad Complutense de Madrid, Madrid, Spain; ^3^Center for Translational Medicine, Temple University School of Medicine, Philadelphia, PA 19140, USA; ^4^Pfizer, CVMED, 610 Main Street, Cambridge, MA, USA; ^5^Instituto de Investigación Sanitaria Gregorio Marañón, Madrid, Spain

## Abstract

Our group has previously demonstrated that short-term treatment with esmolol reduces left ventricular hypertrophy (LVH) in spontaneously hypertensive rats (SHRs). The present study aimed to assess the molecular mechanisms related to this effect. Fourteen-month-old male SHR_s_ were treated intravenously with saline as vehicle (SHR) or esmolol (SHR-E) (300 *μ*g/kg/min). Age-matched vehicle-treated male Wistar-Kyoto (WKY) rats served as controls. After 48 hours of treatment, the hearts were harvested and left ventricular tissue was separated and processed for Western blot analysis to determine the levels of Akt, NF-*κ*B, NFATc4, Creb1, Serca2a, Erk1/2, and Sapk/Jnk. Biomarkers of oxidative stress, such as catalase, protein carbonyls, total thiols, and total antioxidant capacity were evaluated. Esmolol reversed the levels of p-NFATc4, p-Akt, and p-NF-*κ*B in SHRs to the phospholevels of these proteins in WKY rats without modifying p-Erk1/2, p-Sapk/Jnk, p-Creb1, or Serca2a in SHR. Compared with SHR, esmolol increased catalase activity and reduced protein carbonyls without modifying total thiols or total antioxidant capacity. Short-term treatment with esmolol reverses LVH in aged SHRs by downregulation of Akt/NF-*κ*B and NFATc4 activity. Esmolol treatment also increases catalase activity and reduces oxidative stress in SHRs with LVH.

## 1. Introduction

Left ventricular hypertrophy (LVH) is a compensatory response to chronic arterial hypertension. It is an independent risk factor for cardiovascular morbidity and mortality including heart failure, coronary artery diseases, stroke, arrhythmias, and sudden cardiac death [1]. Decrease of LVH is associated with lower cardiovascular risk; therefore, therapies directed towards decreasing LVH are accepted as an essential treatment goal in this condition [2]. Decreases in LVH with antihypertensive therapy (angiotensin-converting enzyme inhibitors, angiotensin receptor blockers, *β*-adrenergic blockers, calcium channel blockers, and diuretics) have been reported, albeit after long-term treatment [3, 4]. Our group reported the first study to show regression of LVH after a markedly short period of treatment with esmolol [5].

Esmolol is an ultrashort-acting cardioselective *β*-adrenergic blocker with a half-life of approximately 2 minutes, a time to peak effect of about 6–10 minutes, and a washout time of 9 minutes [6]. The pharmacodynamic and pharmacokinetic profiles of this drug make it a suitable cardioprotective agent in clinical practice [7, 8].

Our group previously demonstrated that short-term treatment (48 hours) with esmolol reduces LVH in spontaneously hypertensive rats (SHR) [5]. However, the molecular mechanism(s) of this effect have not been elucidated to date. Asymmetrical dimethylarginine (ADMA) is an independent risk factor for the development of LVH. Dimethylarginine dimethyl aminohydrolase (DDAH) plays an important role in the pathogenesis of hypertensive LVH by modulating tissue levels of ADMA [9]. We have previously reported that esmolol reduces ADMA concentrations and increases DDAH concentrations in the left ventricle in SHR model [10, 11]. However, the molecular mechanisms of esmolol-mediated regression of LVH remain unknown.

It is increasingly evident that redox-dependent modifications in cellular proteins and signaling pathways play an important role in cardiac hypertrophy [12]. This can have a significant negative impact on cardiac gene expression that can affect various cellular processes involved in cardiac hypertrophy signaling. A number of transcription factors (e.g., nuclear factor of activated T cells 4 [NFATc4], nuclear factor kappa B [NF-*κ*B], and mitogen-activated protein kinases [MAPKs, e.g., Akt and Erk1/2]) are involved in cardiac hypertrophy signaling [13–15]. The effect of esmolol on the regulation of these transcription factors and MAPKs in LVH signaling remains unknown.

In this study we tested the hypothesis that esmolol could lead to early regression of LVH, at least in part, through MAPK-mediated regulation of NFATc4 and NF-kB transcription factors. Therefore, the present study aimed to assess whether short-term treatment with esmolol can reverse left ventricular hypertrophy in aged SHR hearts and whether these effects are mediated through regulation of Akt/NF-*κ*B and NFATc4.

## 2. Methods

### 2.1. Experimental Animals and Treatment

The study animals: 14-month-old male spontaneously hypertensive rats (SHR) (*n* = 12) and normotensive Wistar-Kyoto (WKY) control rats (*n* = 6) were bred at the animal facility of Universidad Autonoma de Madrid. All rats were supplied with standard rat chow and drinking water ad libitum and were maintained on a 12 h/12 h light/dark cycle. The animals were housed at a constant temperature of 24°C and relative humidity of 40%. Anesthesia was administered by intraperitoneal injection of diazepam 10 mg/kg (Valium 10 mg/ml; Roche Pharmaceuticals, Madrid, Spain) and ketamine 80 mg/kg (Ketolar 50 mg/ml; Parke-Davis, Madrid, Spain), and a catheter was inserted into the right internal jugular vein. SHRs were divided into 2 groups: rats treated with esmolol (SHR-E) and hypertensive control rat group (SHR) treated with saline solution (saline as vehicle). SHR-E received an intravenous infusion of esmolol at 300 *μ*g/kg/min (Breviblock 10 mg/ml; Baxter, Belgium) for 48 hours, and control SHR and WKY received saline solution (vehicle). After 48 hours of treatment, rats were euthanized by decapitation after sedation with an intraperitoneal injection of diazepam 10 mg/kg and ketamine 80 mg/kg. The hearts were harvested within minutes; left ventricles were bisected and processed for Western blot analysis and oxidative stress biomarkers. All procedures fulfilled the stipulations of the Guide for the Care and Use of Laboratory Animals (Directive 2010/63/EU and RD 53/2013) and were approved by the Ethics Committee of Hospital General Universitario Gregorio Marañón, Madrid, Spain.

### 2.2. Systolic Arterial Pressure and Heart Rate Measurements

Systolic arterial pressure and heart rate were measured using the tail-cuff method with a photoelectric sensor (Niprem 546, Cibertec, Madrid, Spain). Several determinations were made at each session before and after treatment (0, 12, 24, 36 and 48 h), and the findings were considered valid if 10 consecutive measurements were within 10 mmHg of each other.

### 2.3. Sample Preparation

Left ventricular tissue samples were homogenized using a TissueLyser LT system (QIAGEN, Hilder, Germany) programmed with 50 s^−1^ oscillation for 4 min in a lysis buffer containing 20 mM Tris-HCl buffer (pH 7.5), 150 mM NaCl, 1 mM EDTA, 1 mM EDTA, 1% Triton X-100, 20 mM sodium orthovanadate, 1 mM sodium fluoride, 1 mM PMSF, and 1% protein inhibitor cocktail acquired from Sigma-Aldrich (Madrid, Spain). Homogenates were centrifuged at 10,000 ×g for 2 min at 4°C and the supernatant was stored at –80°C until analysis.

### 2.4. Western Blot Analysis

Immunoblotting with left ventricular homogenates was used to assess the total (T-) and phosphorylated (p-) levels of NFATc4, AKT, ERK, NF-kB, JNK, CREB, and SERCA. Forty milligrams of left ventricular protein was separated by SDS-PAGE electrophoresis on 12% gel using the Mini-Protean Tetra system (Bio-Rad, Madrid, Spain) at 100 V and room temperature. Separated proteins were transferred onto an Immunoblot PVDF membrane (Bio-Rad, Madrid, Spain) for 1 h at 120 V and 4°C. The efficiency of protein transfer was verified using reversible protein and a stain kit (MemCode, Thermo Scientific, Madrid, Spain). Primary antibodies against total (T) and phosphorylated (p) NFATc4 (Santa Cruz Biotechnology, Germany; dilution factor [DF] = 1 : 200) and p-AKT (DF = 1 : 1000), p-ERK (DF = 1 : 2000), p-NF-*κ*B (DF = 1 : 1000), p-SNP/JNK, p-CREB (DF = 1 : 1000), and SERCA (DF = 1 : 1000) were acquired from Cell Signaling Technology (MA, USA). Primary antibodies were incubated at 4°C overnight. After washing with 1xPBS, the PVDF membrane was incubated with IgG-peroxidase–conjugated secondary anti-rabbit (DF = 1 : 2000) and anti-mouse (DF = 1 : 2000) antibodies (Cell Signaling Technology, USA) overnight at room temperature. Blots were washed again and incubated with the SuperSignal West Pico Chemiluminescent substrate kit (Thermo Scientific, Madrid, Spain). Protein expression bands were acquired with a gel documentation and analysis system (Alliance, Uvitec, Cambridge, UK). Density of the bands on the film was analyzed using the free ImageJ NIH software application. GAPDH antibody (Millipore, Madrid, Spain; DF = 1 : 2000) was used to normalize expression values to correct for protein loading.

### 2.5. Biomarkers of Oxidative Stress

#### 2.5.1. Total Protein Carbonyls

Total protein carbonyls in homogenized left ventricular tissue were quantified using a simplified 2,4-dinitrophenylhydrazine (DNPH) spectrophotometric assay [16], which was adapted to nanovolume. Briefly, 2*μ*L of homogenate was incubated with an equal volume of 10 mM DNPH (in 2.5 N HCl) at room temperature for 10 min, and 1*μ*L sodium hydroxide (6 N) was then added to the mixture, which was incubated again for 10 min at room temperature. Absorbance at 450 nm was read immediately in a Nanodrop 2000 spectrophotometer (Thermo Scientific, NC, USA). Protein carbonyl levels were calculated using the extinction coefficient of DNPH at 450 nm (*ε* = 22308 M^−1^ cm^−1^) and optical path length of 1 mm and expressed as nmol of carbonyls/mg protein. The time of incubation of samples after addition of sodium hydroxide was synchronized so that it was 10 min for all samples, because DNPH is unstable under alkaline conditions. The total protein of homogenates was assessed using Bradford reagent according to the manufacturer's recommendations (Bio-Rad, USA).

#### 2.5.2. Total Thiols

Left ventricular total thiol levels were assessed using the 5,5′-dithiobis (2-nitrobenzoic acid) assay [17] adapted to nanovolume. Absorbance was measured at 412 nm in a Nanodrop 2000 spectrophotometer (Thermo Scientific, NC, USA), and total thiol content was expressed as millimoles per liter of reduced glutathione per milligram of protein.

#### 2.5.3. Total Antioxidant Capacity (TAC)

Total antioxidant capacity of left ventricular homogenates was assessed using the CUPRAC-BCS assay [18] adapted to nanovolume. Absorbance at 490 nm was read in a Nanodrop 2000 spectrophotometer (Thermo Scientific, NC, USA). Total antioxidant capacity values were obtained from the standard curve of the antioxidant trolox (0–2 mol/L) and expressed as *μ*mol/L Trolox.

#### 2.5.4. Catalase Activity

Catalase activity was assessed using the Amplex Red assay (Invitrogen, Spain) adapted to a 384-well plate. Absorbance at 560 nm was read after 30 min of reaction at room temperature in a multimode microplate reader (Synergy HT, Bio-Tek, VT, USA). Catalase activity was expressed as U/mg protein, calculated from the CAT standard curve (0–2 U/mL).

#### 2.5.5. Protein Content

Protein content was assessed using the Coomassie blue-based microtiter plate assay according to the manufacturer's recommendations (Bio-Rad, Madrid, Spain). Absorbance was measured at 595 nm in a Synergy HT Multimode Microplate Reader (Bio-tek), and bovine serum albumin was used as the standard.

### 2.6. Statistical Analysis

The results were expressed as the mean ± SEM. The parameters were compared using repeated measures analysis of variance (physiological parameters) or single-factor (rat) analysis of variance (western blot analysis and oxidative stress parameters). A post hoc Bonferroni correction was applied. Statistical significance was set at *P* ≤ 0.05. The analysis was performed using IBM SPSS Statistics for Windows, version 20.0 (IBM Corp, Armonk, New York, USA) and Prism GraphPad 6.0 (GraphPad Software, California, USA).

## 3. Results

### 3.1. Blood Pressure and Heart Rate Measurements

Values of physiological parameters are shown in Table 1. Systolic arterial pressure was significantly higher in SHR than in WKY controls (*P* < 0.01). Short-term administration of esmolol for 48 hours significantly reduced systolic arterial pressure in SHR-E during the 48 h of treatment, and these values were comparable to those of the WKY. No statistically significant differences in heart rate were found between WKY and SHR, and heart rate was reduced in SHR-E during the 48 h of treatment with respect to WKY (*P* < 0.001) and SHR (*P* < 0.001).

### 3.2. Short-Term Administration of Esmolol Decreases NFATc4 Phosphorylation in the Left Ventricular Myocardial Tissue of Adult Spontaneously Hypertensive Rats

**Figure 1:**
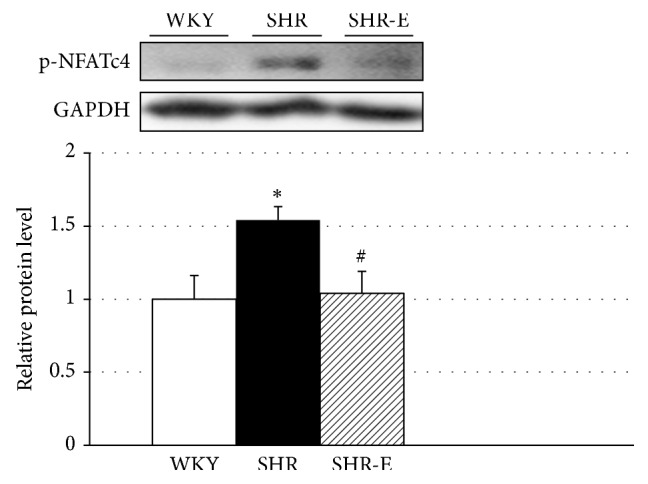
Expression levels of p-NFATc4 in the left ventricle. Experiments were performed in homogenates of left ventricular tissue from Wistar-Kyoto rats (WKY), spontaneously hypertensive rats (SHR), and esmolol-treated SHRs (SHR-E). Values are shown as mean ± SEM; *n* = 6. ^*∗*^*P* < 0.05 versus WKY and ^#^*P* < 0.05 versus untreated SHR.

**Figure 2:**
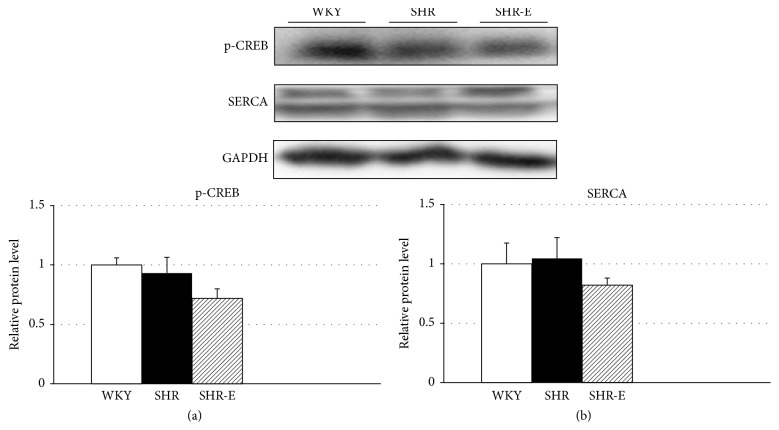
Expression levels of p-Creb1 and Serca2a in the left ventricle. Experiments were performed in homogenates of left ventricular tissue from Wistar-Kyoto rats (WKY), spontaneously hypertensive rats (SHR), and esmolol-treated SHRs (SHR-E). Values are shown as mean ± SEM; *n* = 6.

In the whole tissue left ventricular homogenates, p-NFATc4 levels were markedly higher in SHR than in WKY controls, and short-term treatment of SHR with esmolol significantly reduced p-NFATc4 levels (Figure 1, *P* < 0.05). Serca2a and p-Creb1 expression in the left ventricle of untreated and treated rats were similar (Figures 2(a) and 2(b)). This finding may suggest a decrease in the cardiac hypertrophy signaling in adult SHR.

### 3.3. Short-Term Administration of Esmolol Decreases Akt and NF-kB Phosphorylation in the Left Ventricle of Adult SHR

**Figure 3:**
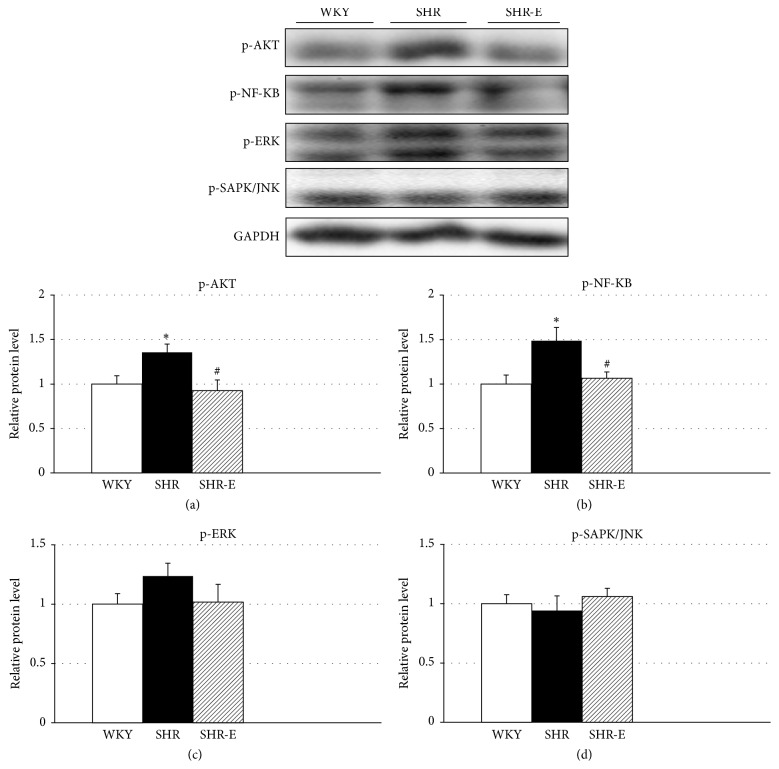
Expression levels of p-Akt (a), p-NF-*κ*B (b), p-Erk1/2 (c), and p-Sapk/Jnk (d) in left ventricular tissue. Experiments were performed using left ventricular homogenates from Wistar-Kyoto rats (WKY), spontaneously hypertensive rats (SHR), and esmolol-treated SHRs (SHR-E). Values are shown as mean ± SEM; *n* = 6. ^*∗*^*P* < 0.05 versus WKY and ^#^*P* < 0.05 versus untreated SHR.

p-Akt and p-NF-kB expression in the left ventricular tissue was lower in SHR-E than in SHR and treatment with esmolol reduced this expression to the protein levels of WKY (Figures 3(a) and 3(b)). p-Akt levels were higher in SHR versus WKY (Figure 3(a), *P* < 0.05). p-NF-kB levels were higher in SHR versus WKY (Figure 3(b), *P* < 0.05). No differences in p-Sapk/Jnk expression were observed when comparing the three experimental groups (Figure 3(d)). There was a small trend in p-Erk1/2 increase in the SHR group but it was not statistically significant (Figure 3(c)).

### 3.4. Short-Term Administration of Esmolol Reduces Oxidative Stress and Increases Catalase Activity in the Left Ventricular Tissue

**Figure 4:**
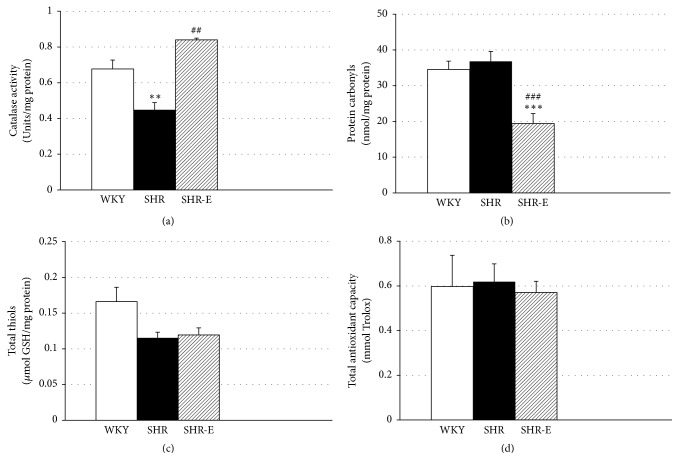
Left ventricular oxidative stress biomarkers: catalase activity (a), protein carbonyls (b), total thiols (c), and total antioxidant capacity (d). Experiments were performed in left ventricular homogenates from Wistar-Kyoto rats (WKY), spontaneously hypertensive rats (SHR), and esmolol-treated SHRs (SHR-E). Values are shown as mean ± SEM; *n* = 6. ^*∗∗*^*P* < 0.01 versus WKY, ^*∗∗∗*^*P* < 0.001 versus WKY and ^##^*P* < 0.01 versus untreated SHR, and ^###^*P* < 0.001 versus untreated SHR.

Short-term treatment with esmolol restored catalase activity to the levels in WKY control heart tissue. Compared to WKY rats and SHR-E, catalase activity was decreased in SHR animals (Figure 4(a), *P* < 0.01). Protein carbonyl levels in the left ventricular tissue of SHR-E were lower than in SHR and WKY rat hearts (Figure 4(b),* P* < 0.05), and comparable in SHR and WKY (Figure 4(b)). No differences were observed in total thiols and total antioxidant capacity in the three experimental groups (Figures 4(c) and 4(d)).

## 4. Discussion

We previously reported that short-term treatment with esmolol produces early regression of left ventricular hypertrophy (LVH) in SHR [5]. Esmolol led to early reduction of left ventricular mass, early changes in the cross-sectional area of left ventricular cardiomyocytes, and a marked decrease in glucose metabolism in the hypertrophied ventricle [5]. We recently showed that esmolol reduces ADMA concentrations and increases DDAH concentrations in the left ventricle in SHR [10, 11]. These findings would explain, in part, the regression of LVH, although several mechanisms could be involved in regression of LVH with esmolol. In the present study we show that short-term administration of esmolol reversed LVH in adult SHR at least in part by downregulation of Akt/NF-*κ*B and NFATc4 protein phosphorylation and reduced oxidative stress. It also increases catalase activity in the left ventricular tissue.

Induction of the calcineurin-NFAT pathway is a common hallmark of pathological hypertrophy [19–21]. Calcineurin is a calcium/calmodulin–activated serine/threonine phosphatase that once stimulated de-phosphorylates and thereby activates the NFAT transcription factor. Dephosphorylated NFAT translocate from the cytoplasm to the nucleus which leads to the transcription of cardiac hypertrophy-associated genes [19–21]. We found that p-NFATc4 was markedly higher in left ventricular heart tissue from SHR compared to WKY. NFATc4, one of five NFAT family members, was shown to be capable of promoting cardiac hypertrophy in vivo. NFATc4 functions as a calcineurin effector by trafficking between the cytosol and the nucleus to regulate the cardiac hypertrophic response [22]. Although p-NFATc4 levels in cytoplasm are decreased as a consequence of nuclear translocation in cardiomyocytes, studies on protein expression in left ventricular whole heart tissue in hypertrophic animal models are scarce. NFATc4 has been described as a new mechanism that contributes to the induction of pathological cardiac hypertrophy [22] Interestingly, short-term treatment of SHR with esmolol significantly reduced p-NFATc4 levels.

Esmolol is an unique cardioselective *β*1-receptor blocking agent with a rapid onset and short duration of action that has been tested in a variety of patients, including those with unstable angina, myocardial ischemia, supraventricular arrhythmias, and peri- and postoperative tachycardia and hypertension [6–8]. In surgical and critical care setting where clinical conditions are rapidly changing, the pharmacokinetic profile of esmolol (it has a very short half-life and a short duration of action) allows the drug to provide rapid pharmacologic control and minimizes the potential for serious adverse effects. A meta-analysis of 67 randomized clinical trials shows that esmolol effectively decreased both heart rate and arterial blood pressure [8]. Intravenous esmolol has been shown to reduce the incidence of myocardial ischemia and arrhythmias in cardiac (a total of 778 patients from 20 randomized trials) and noncardiac surgery (a total of 1765 patients from 32 randomized trials) without increasing the episodes of hypotension and bradycardia [23, 24]. One proposed benefit of *β*-receptor blockade is a myocyte-autonomous reduction in hypertrophy and cell death [21]. Activating *β*-adrenergic receptors for extended periods of time (hypertension, heart failure, or volume overload due to valve dysfunction) leads to hypertrophy and *β*-receptor desensitization [21, 25]. Furthermore, the age-related reduction in cardiac *β*-adrenergic receptor sensitivity and density has been reported in the myocardium [6]. In the present study, *β*-adrenergic receptor desensitization was demonstrated by the similar expression of Serca2a and p-Creb1 observed in the left ventricle of untreated and treated aged SHR [21]. A previous study has also shown that SHR and WKY rats showed similar Serca2a levels [26, 27]. Therefore, short-term treatment with esmolol can regulate cardiac hypertrophy in adult SHR through regulation of molecular pathways other than *β*-adrenergic blockade, as suggested elsewhere [21].

Calcineurin-NFAT and PI3K/Akt signaling pathways are interdependent and together orchestrate the cardiac hypertrophic response [21]. The NF*κ*B signaling cascade interacts with PI3K/Akt signaling pathways [28]. We found that levels of p-Akt in the left ventricular tissue were lower in SHR-E than in SHR. Upregulation of Akt can contribute to adaptive cardiac hypertrophy; however, persistent Akt activation over longer period of time can lead to unsustainable cardiac hypertrophy signaling that may develop into decompensation leading to heart failure [29]. In contrast to our findings, levels of p-Akt were reported to be lower for SHR than for normotensive WKY [30, 31]. Similarly, esmolol reduced p-NF-*κ*B expression to the protein level of WKY. NF-*κ*B has also been postulated to play a role in cardiac hypertrophy [20, 32].

The Akt pathway is actively involved in the regulation of NF-*κ*B [33]. Akt can activate NF-*κ*B by inducing phosphorylation [28], and NF-*κ*B inhibition attenuates cardiac hypertrophy [34]. A recent study demonstrated that NFATc4 and NF-*κ*B could interact and assemble a transcriptional complex that effectively coordinates cardiac hypertrophy and pathological remodeling [35]. Thus, it was recently observed that the nuclear translocation of NFATc4 and the transcriptional activity of NF-*κ*B were significantly increased by phenylephrine, which was significantly inhibited by pretreatment with the known antioxidant alpha-lipoic acid [36].

In a previous study, our group has demonstrated that, compared with SHR, plasma superoxide anion scavenging activity (including superoxide dismutase) was more pronounced in SHR-E and this was associated with an improvement in nitric oxide availability measured as nitrites/nitrates [37]. In the present study, short-term administration of esmolol restored catalase activity to the level detected in WKY controls. Catalase activity was less pronounced in SHR than in WKY and SHR-E, thus reflecting the possibility that esmolol also increases superoxide dismutase activity in the left ventricular tissue because an increase in hydrogen peroxide may induce catalase activity. Indeed, catalase activity in blood is a good indicator of the redox status of the heart [38]. NADPH oxidase (Nox2) was found to be essential for angiotensin II–induced cardiac hypertrophy [39], which is involved in the enhanced activation of MAPKs and the NF-*κ*B pathway [12]. Protein carbonyl levels were also lower in the left ventricular tissue of SHR-E, although they remained unchanged in untreated SHR and WKY. Finally, similar to findings from our previous study based on plasma from SHR-E [37], no differences in total antioxidant capacity or total thiols were observed between the three experimental groups.

Left ventricular hypertrophy (LVH) is probably the most visible manifestation of hypertensive organ damage [1]. Several clinical and experimental animal studies have shown that cardiac hypertrophy is reversed by various antihypertensive drugs (angiotensin-converting enzyme inhibitors, angiotensin receptor blockers, *β*-adrenergic blockers, calcium channel blockers, and diuretics), although they were based on long-term therapy. On the other hand, a better understanding of the cellular and molecular mechanisms underlying cardiac hypertrophy is critical when attempting to identify new therapeutic targets to inhibit LVH. We report the results of the first study to associate regression of LVH after a markedly short administration, 48 hours, of a *β*-blocker esmolol [5]. However, it is necessary to study in more detail the underlying molecular mechanisms of positive effect of esmolol on inhibition of LVH before testing this treatment modality/regimen in humans. If these effects of short esmolol administration could be confirmed in humans, esmolol could be an option for the treatment of LVH in critical care units.

In conclusion, our findings show that short-term administration of esmolol reverses LVH in adult SHR through inhibition of Akt/NF-*κ*B and NFATc4 phosphorylation, reduction of oxidative stress, and increased catalase activity in the left ventricular cardiac tissue.

## Figures and Tables

**Table 1:** Systolic blood pressure and heart rate in WKY, SHR, and SHR-E.

Groups	Time treatment (hours)	SBP (mmHg)	HR (bpm)
WKY	0	126 ± 21	310 ± 20
	12	135 ± 18	317 ± 15
	24	119 ± 11	324 ± 21
	36	121 ± 22	319 ± 19
	48	127 ± 23	320 ± 12

SHR	0	190 ± 20_ _^*∗∗*^	322 ± 15
	12	217 ± 25_ _^*∗∗*^	311 ± 11
	24	198 ± 21_ _^*∗∗*^	324 ± 18
	36	223 ± 12_ _^*∗∗∗*^	312 ± 20
	48	205 ± 23_ _^*∗∗*^	313 ± 16

SHR-E	0	199 ± 31_ _^*∗∗*^	320 ± 13
	12	137 ± 15_ _^##^	229 ± 12_ _^*∗∗∗*, ###^
	24	139 ± 28_ _^##^	231 ± 10_ _^*∗∗∗*, ###^
	36	145 ± 11_ _^##^	219 ± 18_ _^*∗∗∗*, ###^
	48	143 ± 10_ _^##^	220 ± 13_ _^*∗∗∗*, ###^

WKY: Wistar-Kyoto rats; SHR: spontaneously hypertensive rats; SHR-E: esmolol-treated SHR rats; SBP: systolic blood pressure; HR: heart rate. Values are shown as mean ± SEM. *n* = 6.^*∗∗*^*P* < 0.01 versus WKY and ^*∗∗∗*^*P* < 0.001 versus WKY; ^##^*P* < 0.01 versus untreated SHR and ^###^*P* < 0.001 versus untreated SHR.
